# L-Carnitine Is Involved in Hyperbaric Oxygen-Mediated Therapeutic Effects in High Fat Diet-Induced Lipid Metabolism Dysfunction

**DOI:** 10.3390/molecules25010176

**Published:** 2020-01-01

**Authors:** Junhua Yuan, Qixiao Jiang, Limin Song, Yuan Liu, Manwen Li, Qian Lin, Yanrun Li, Kaizhen Su, Zhengye Ma, Yifei Wang, Defeng Liu, Jing Dong

**Affiliations:** 1 Department of Specialty Medicine, School of Basic Medicine, Qingdao University, Qingdao 266071, China; yuanjunhua322@126.com (J.Y.); songliminn@163.com (L.S.); yuanliu_qdu2017@163.com (Y.L.); limanwen615@163.com (M.L.); linqian0921@163.com (Q.L.); 2 Department of Toxicology, School of Public Health, Qingdao University, Qingdao 266071, China; 3 Department of Clinical Medicine, Medical Collage, Qingdao University, Qingdao 266071, China; liyanrun98@126.com (Y.L.); sukaizhen1998@163.com (K.S.); maaazy@163.com (Z.M.); wyffighting@126.com (Y.W.); Liudefeng928@163.com (D.L.); 4 Department of Physiology, School of Basic Medicine, Qingdao University, Qingdao 266071, China

**Keywords:** hyperbaric oxygen, high fat diet, lipid metabolism dysfunction, L-carnitine, PPARα, CPT1b

## Abstract

Lipid metabolism dysfunction and obesity are serious health issues to human beings. The current study investigated the effects of hyperbaric oxygen (HBO) against high fat diet (HFD)-induced lipid metabolism dysfunction and the roles of L-carnitine. C57/B6 mice were fed with HFD or normal chew diet, with or without HBO treatment. Histopathological methods were used to assess the adipose tissues, serum free fatty acid (FFA) levels were assessed with enzymatic methods, and the endogenous circulation and skeletal muscle L-carnitine levels were assessed with liquid chromatography-tandem mass spectrometry (LC-MS/MS). Additionally, western blotting was used to assess the expression levels of PPARα, CPT1b, pHSL/HSL, and UCP1. HFD treatment increased body/adipose tissue weight, serum FFA levels, circulation L-carnitines and decreased skeletal muscle L-carnitine levels, while HBO treatment alleviated such changes. Moreover, HFD treatment increased fatty acid deposition in adipose tissues and decreased the expression of HSL, while HBO treatment alleviated such changes. Additionally, HFD treatment decreased the expression levels of PPARα and increased those of CPT1b in skeletal muscle, while HBO treatment effectively reverted such changes as well. In brown adipose tissues, HFD increased the expression of UCP1 and the phosphorylation of HSL, which was abolished by HBO treatment as well. In summary, HBO treatment may alleviate HFD-induced fatty acid metabolism dysfunction in C57/B6 mice, which seems to be associated with circulation and skeletal muscle L-carnitine levels and PPARα expression.

## 1. Introduction

Obesity is a global health issue, affecting both developed and developing countries. Due to the increasing consumption of higher energy diets as well as lower energy expenditure, the prevalence of obesity is increasing remarkably throughout the world [1]. According to the world health organization (WHO), the global prevalence of obesity almost doubled from 1980 to 2008, and the prevalence of general obesity in China increased by about 90% [2], which is now attracting attention.

Generally, obesity is defined as a chronic pathological condition characterized by excess fat deposition in adipose tissue, accompanied by metabolic dysfunctions, including increased lipid storage in other tissues [3], compensatory lipolysis increase [4], fatty acid oxidation dysfunction in skeletal muscle [5] and increased circulatory free fatty acid (FFA) [6]. These lipid metabolism dysfunctions are alarming indicators of type 2 diabetes, cardiovascular disease, and even cancer [7,8]. The underlying molecular mechanism is currently being extensively studied, but much still needs to be done.

Skeletal muscle plays important roles in fatty acid metabolism [9], especially in the oxidative slow-twitch fibers [10]. High fat diet (HFD) has been shown to induce compensatory increase in skeletal muscle mitochondrial biogenesis and beta-oxidation [11], while mitochondrial complication and interrupted beta-oxidation was also reported as an outcome of HFD treatment [12]. Many molecules are involved in the process of beta-oxidation. L-carnitine, a quaternary amine molecule, is especially important since it has multiple roles in fatty acid metabolism. The major role of L-carnitine is participation of the classical “carnitine shuttle” process [13]. Changes in the levels of L-carnitine had been associated with expression levels of CPT1 and beta oxidation levels [14], suggesting that the level of L-carnitine is associated with the efficacy of beta-oxidation. Additionally, it has also been demonstrated that a decreased L-carnitine level in skeletal muscle is associated with increased circulatory FFA: The latter is mainly released from adipose tissue via lipolysis, in which hormone-sensitive lipase (HSL) is the rate limiting enzyme [15], indicating that L-carnitine may affect lipid metabolism in both skeletal muscle and the major fatty acid storage, adipose tissue. Notably, contradictive results were present regarding to L-carnitine’s effects on obesity and metabolic syndrome. While it is generally accepted that L-carnitine supplement is not effective in weight loss or correcting dyslipidemia [16], evidences supporting its effect on obesity and improving lipid metabolism were also reported [17,18]. It is necessary to further investigate the role of L-carnitine in fatty acid metabolism dysfunction.

Many methods have been administered for management of fatty acid metabolism dysfunction and obesity, such as pharmaceutical agents [19] and physical activities [20]. However, low response rates and adverse effects [21] limited their application, thus new potential therapies for fatty acid metabolism dysfunction and obesity are highly desirable. Hyperbaric oxygen (HBO) therapy is a physical therapy, in which 100% oxygen with higher than 1 atmosphere absolute was administered to the patients. Clinically, HBO was applied for treatment of decompression sickness, carbon monoxide poisoning, clostridial infections, diabetic dermal complications, burning, radiation damage, and delayed wound healing [22]. Recently, it has also been shown that HBO treatment may help with metabolic syndromes [23]. Thus, in the current study, HBO treatment was applied along with HFD in C57/B6 mice to determine whether it may alleviate HFD-induced fatty acid metabolism dysfunction.

The main physiological effect of HBO treatment is to provide high levels of oxygen, promoting cell respiration as well as oxidation [24]. However, to the best of our knowledge, no report exists for its effect on the endogenous L-carnitine levels. Considering the central role of L-carnitine in fatty acid metabolism and beta oxidation, it is highly likely that it is involved in HBO’s protective effects. Endogenous circulatory and skeletal levels of L-carnitine were determined with liquid chromatography-tandem mass spectrometry (LC-MS/MS) in the current study, further revealing the role of L-carnitine in HBO mediated protective effects.

In the current study, an HFD-induced fatty acid metabolism dysfunction model was established in C57/B6 mice, in which the potential protective effects of HBO treatment were assessed, and the role of endogenous L-carnitine in the fatty acid metabolism dysfunction and HBO mediated alleviation investigated. Furthermore, peroxisome proliferator-activated receptor alpha (PPARα), the major regulator of beta oxidation [25] and its downstream gene, CPT1b, the rate limiting enzyme in the carnitine palmitoyltransferase system were explored as potential underlying molecular mechanism. Our data added to the knowledge base of fatty acid metabolism dysfunction, and provided evidence for applying HBO treatment as a potential therapy for fatty acid metabolism dysfunction and obesity.

## 2. Results

### 2.1. Experimental Design and General Parameters

**Figure 1:**
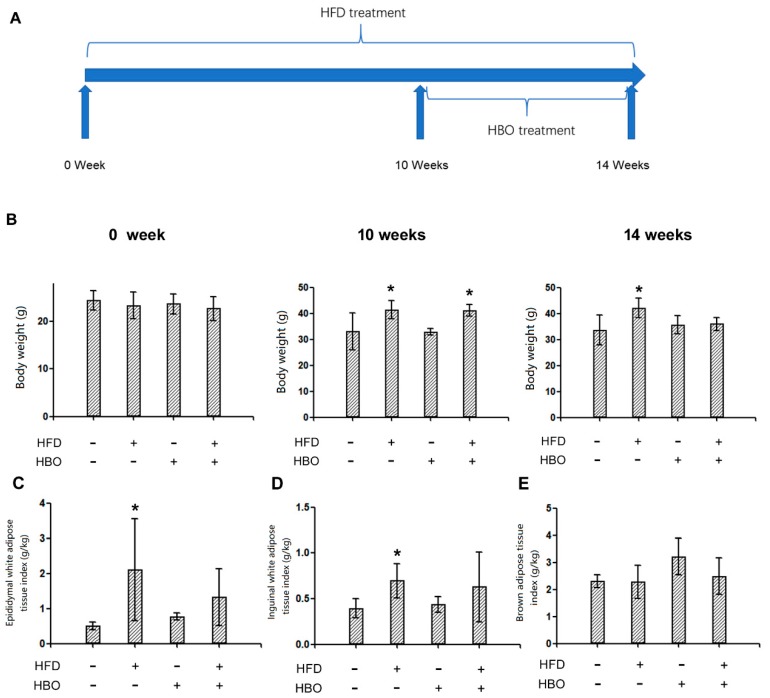
Experimental design, whole body, epididymal white adipose tissue (EWAT), inguinal white adipose tissue (IWAT), and brown adipose tissue (BAT) weight. C57/B6 mice were kept in 21–25 °C environment with 12 h light/dark cycle. Food and water were provided ad labium. Animals received normal chew diet, or HFD (15% lard, 3% soybean oil, 5% egg yolk, 18% sugar, and 59% chew diet) for a total of 14 weeks. HBO treatment (5 min pressure rise stage, 60 min stabilization stage with 2.0 atmospheres absolute and 100% oxygen, 5 min depression stage) were applied during the last 4 weeks of diet treatment. At the end of treatments, whole body weight was measured, then animals were sacrificed, EWAT, IWAT, BAT, and skeletal muscle were weighed and collected. Three to five animals were included in each group. Error bars represent standard derivation. (**A**): The whole experimental design. (**B**): The body weights at 0-, 10- and 14-week. (**C**): EWAT weight at the end of study. (**D**): IWAT weight at the end of study. (**E**): BAT weight at the end of study. *: statistically different from control group animals (*p* < 0.05).

The timeline design of the experiments was reported in Figure 1A. After 0, 10, or 14 weeks of treatments, the body weights were reported in Figure 1B. 10 or 14 weeks of HFD treatment resulted in significantly higher body weight of the animals than those received normal diet, indicating successful establishment of HFD-induced obesity model. On the other hand, HBO co-treatment for 4 weeks effectively abolished such changes, suggesting protective effects of HBO. The epididymal white adipose tissue (EWAT), inguinal white adipose tissue (IWAT), and brown adipose tissue (BAT) weights were reported in Figure 1C,D,E, respectively. The EWAT and IWAT weights significantly changed in a similar pattern as the body weight, while no significant changes were observed in the BAT weight.

### 2.2. Histological Assessments

#### 2.2.1. Hematoxylin and Eosin Staining for EWAT

**Figure 2:**
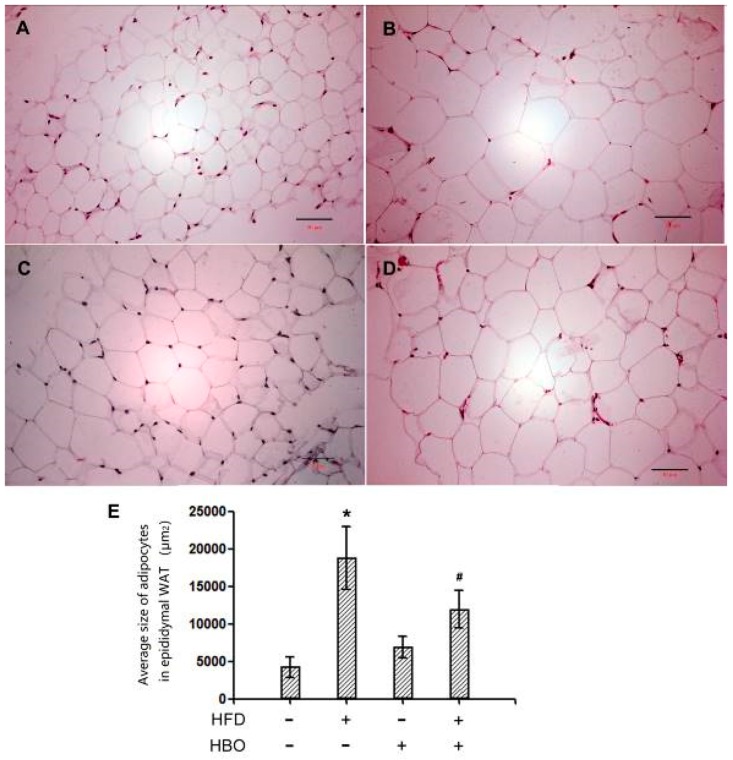
Hematoxylin and eosin staining of EWAT. EWAT tissues were fixed in 4% paraformaldehyde for 24 h, and then histologically processed and sectioned at thickness of 6 um. Hematoxylin and eosin staining was performed following manufacturer’s instructions. Quantification was performed with ImageJ. Three samples from three independent animals were assessed per group. Error bars represent standard derivation. Scale bars represent 50 um. (**A**): Representative hematoxylin and eosin staining picture of EWAT from control animals. (**B**): Representative hematoxylin and eosin staining picture of EWAT from HFD-treated animals. (**C**): Representative hematoxylin and eosin staining picture of EWAT from HBO-treated animals. (**D**): Representative hematoxylin and eosin staining picture of EWAT from HFD + HBO-treated animals. (**E**): Quantification of the adipocyte areas. *: statistically different from control group animals (*p* < 0.05). #: statistically different from HFD group animals (*p* < 0.05).

Representative hematoxylin and eosin staining pictures for EWAT were shown in Figure 2A–D. Quantification for the average adipocyte size was reported in Figure 2E. It was revealed that the adipocyte size significantly increased in the samples from HFD-treated animals, while HBO treatment may effectively alleviate such changes.

#### 2.2.2. Hematoxyin and Eosin Staining for BAT

**Figure 3:**
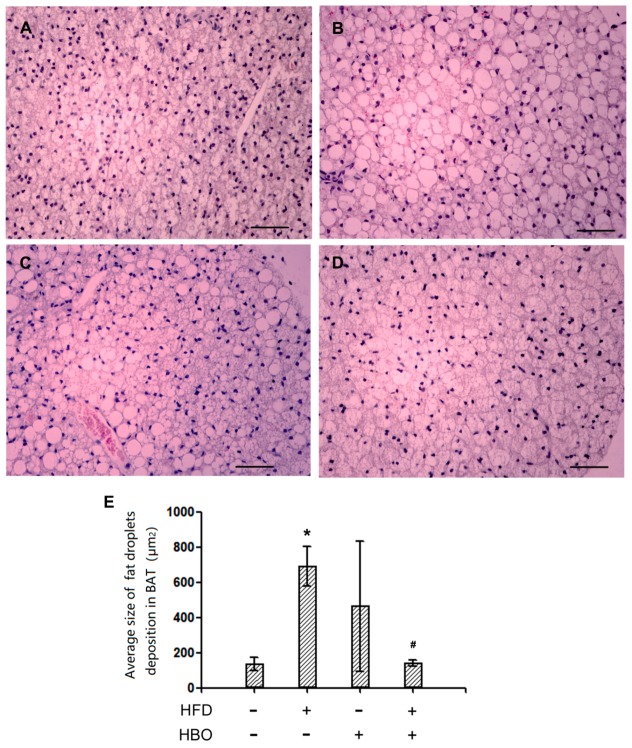
Hematoxylin and eosin staining of BAT. BAT tissues were fixed in 4% paraformaldehyde 24 h, and then histologically processed and sectioned at thickness of 6 um. Hematoxylin and eosin staining was performed following manufacturer’s instructions. Quantification was performed with ImageJ. Three samples from three independent animals were assessed per group. Error bars represent standard derivation. Scale bars represent 50 um. (**A**): Representative hematoxylin and eosin staining picture of BAT from control animals. (**B**): Representative hematoxylin and eosin staining picture of BAT from high fat diet (HFD)-treated animals. (**C**): Representative hematoxylin and eosin staining picture of BAT from HBO-treated animals. (**D**): Representative hematoxylin and eosin staining picture of BAT from HFD + HBO-treated animals. (**E**): Quantification of the average size of fat droplets. *: statistically different from control group animals (*p* < 0.05). #: statistically different from HFD group animals (*p* < 0.05).

Representative hematoxylin and eosin staining pictures for BAT were shown in Figure 3A–D. Quantification for the average size of fat droplets was reported in Figure 3E. It was revealed that the size of fat droplets significantly increased in the samples from HFD-treated animals, while HBO treatment may effectively alleviate such changes.

#### 2.2.3. Immunohistochemistry for HSL in EWAT

**Figure 4:**
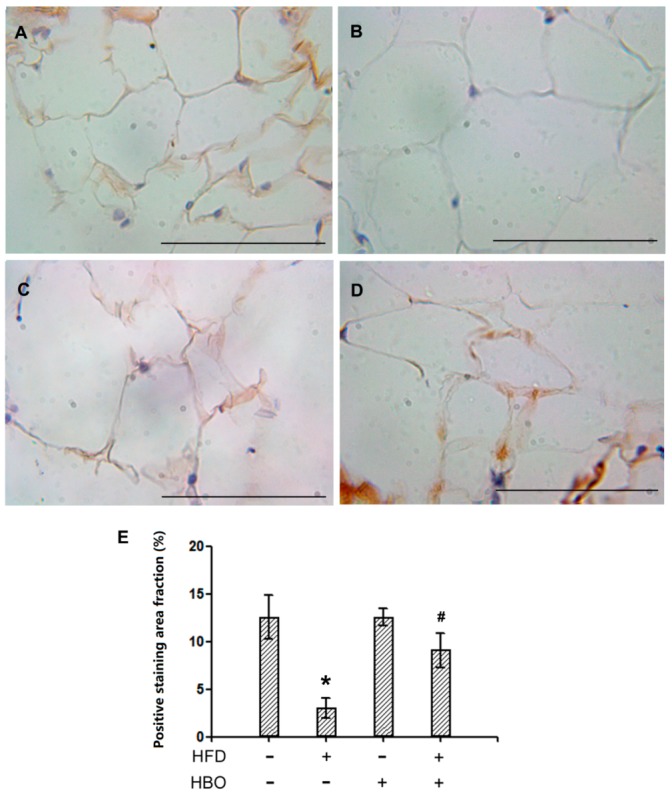
Immunohistochemistry for hormone-sensitive lipase (HSL) on EWAT. EWAT tissues were fixed in 4% paraformaldehyde for 24 h, and then histologically processed and sectioned at thickness of 6 um. Immunohistochemistry for HSL was performed following manufacturer’s instructions. Quantification was performed with ImageJ. Three samples from three independent animals were assessed per group. Error bars represent standard derivation. Scale bars represent 70 um. (**A**): Representative immunohistochemistry picture of EWAT from control animals. (**B**): Representative immunohistochemistry picture of EWAT from HFD-treated animals. (**C**): Representative immunohistochemistry picture of EWAT from HBO-treated animals. (**D**): Representative immunohistochemistry picture of EWAT from HFD + HBO-treated animals. (**E**): Quantification of the positive staining fraction. *: statistically different from control group animals (*p* < 0.05). #: statistically different from HFD group animals (*p* < 0.05).

Representative immunohistochemistry pictures for HSL in EWAT were shown in Figure 4A–D. Quantification for the positively stained area percentages were reported in Figure 4E. The results indicated that the expression levels of HSL were significantly decreased in samples from HFD-treated animals, while HBO treatment effectively alleviated such changes. Decreased expression of the rate limiting lipolysis enzyme HSL indicated that HFD decreased the capacity of lipolysis in EWAT, which might be contributing to the observed larger adipocyte size.

#### 2.2.4. Immunohistochemistry for HSL in BAT

**Figure 5:**
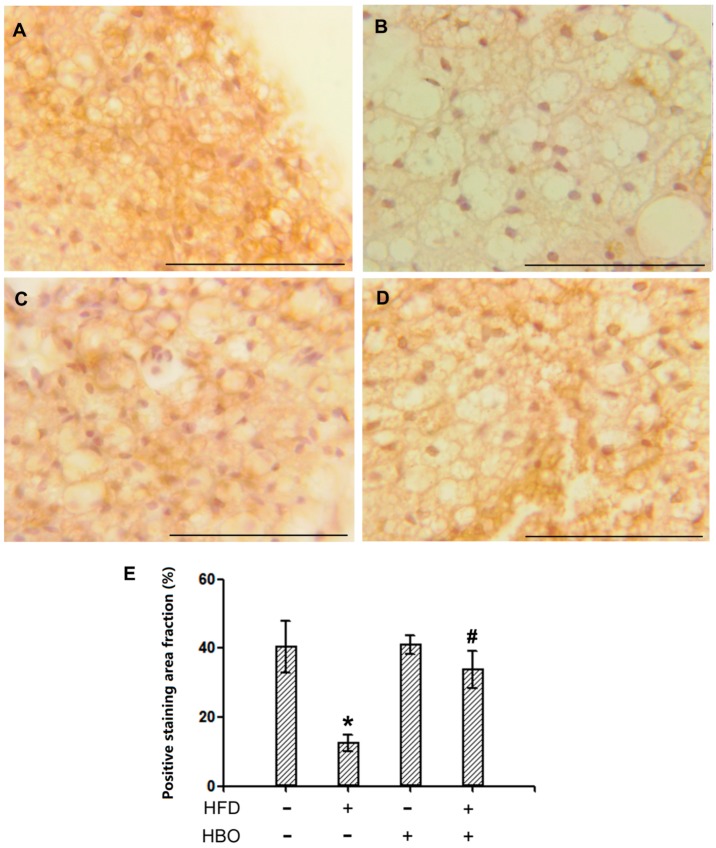
Immunohistochemistry for HSL on BAT. BAT tissues were fixed in 4% paraformaldehyde 24 h, and then histologically processed and sectioned at thickness of 6 um. Immunohistochemistry for HSL was performed following manufacturer’s instructions. Quantification was performed with ImageJ. Three samples from three independent animals were assessed per group. Error bars represent standard derivation. Scale bars represent 70 um. (**A**): Representative immunohistochemistry picture of BAT from control animals. (**B**): Representative immunohistochemistry picture of BAT from HFD-treated animals. (**C**): Representative immunohistochemistry picture of BAT from HBO-treated animals. (**D**): Representative immunohistochemistry picture of BAT from HFD + HBO-treated animals. (**E**): Quantification of the positive staining fraction. *: statistically different from control group animals (*p* < 0.05). #: statistically different from HFD group animals (*p* < 0.05).

Representative immunohistochemistry pictures for HSL in BAT were shown in Figure 5A–D. Quantification for the positively stained area percentages were reported in Figure 5E. The results indicated that the expression levels of HSL were significantly decreased in samples from HFD-treated animals, while HBO treatment effectively alleviated such changes.

### 2.3. Serum FFA Levels

**Figure 6:**
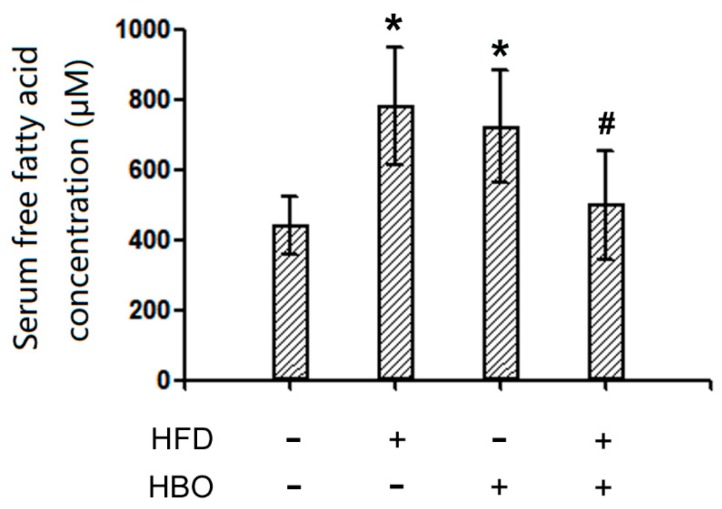
Serum free fatty acid (FFA) levels. Serum FFA levels were measured with a commercially available kit (BC0595, Solarbio, China). Three to five samples from independent animals were included in the tests. Error bars represent standard derivation. *: statistically different from control group animals (*p* < 0.05). #: statistically different from HFD group animals (*p* < 0.05).

Serum FFA levels were reported in Figure 6. Following HFD treatment, the serum FFA levels remarkably increased, while HBO treatment effectively abolished such changes. Notably, HBO solo treatment also led to increased serum FFA levels.

### 2.4. Endogenous L-Carnitine Levels in Serum and Skeletal Muscle

**Figure 7:**
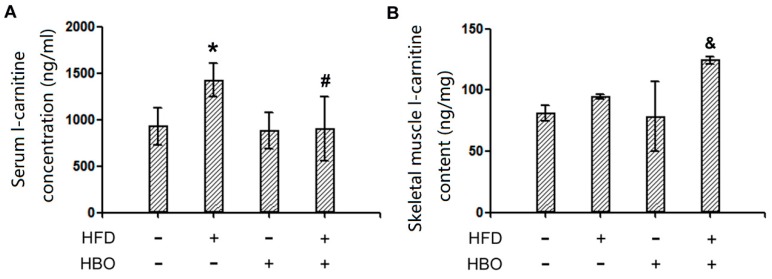
Serum and skeletal muscle L-carnitine levels. Serum and skeletal muscle L-carnitine levels were measured with LC-MS/MS. Please refer to supplementary materials for method and quality control details. Three to five samples from independent animals were included in the tests. Error bars represent standard derivation. (**A**): Serum L-carnitine levels. (**B**): Skeletal muscle L-carnitine levels. *: statistically different from control group animals (*p* < 0.05). #: statistically different from HFD group animals (*p* < 0.05). &: statistically different from HBO group animals (*p* < 0.05).

LC-MS/MS results of serum and skeletal muscle L-carnitine levels were reported in Figure 7 Interestingly, differential patterns were observed: L-carnitine levels remarkably increased in the serum of HFD treated animals and HBO treatment abolished such changes (Figure 7A). On the other hand, no statistical differences were observed in the L-carnitine levels in the skeletal muscle of HFD treated animals, while HBO treatment significantly increased the levels of L-carnitine (Figure 7B). The data suggested that HFD changed the distribution of L-carnitine in the system, increasing circulatory levels, but not the skeletal muscle levels while HBO treatment returned circulatory levels of L-carnitine to normal, but dramatically increased skeletal muscle levels of L-carnitine.

### 2.5. Western Blotting for pHSL/HSL and UCP1 in Brown Adipose Tissues

**Figure 8:**
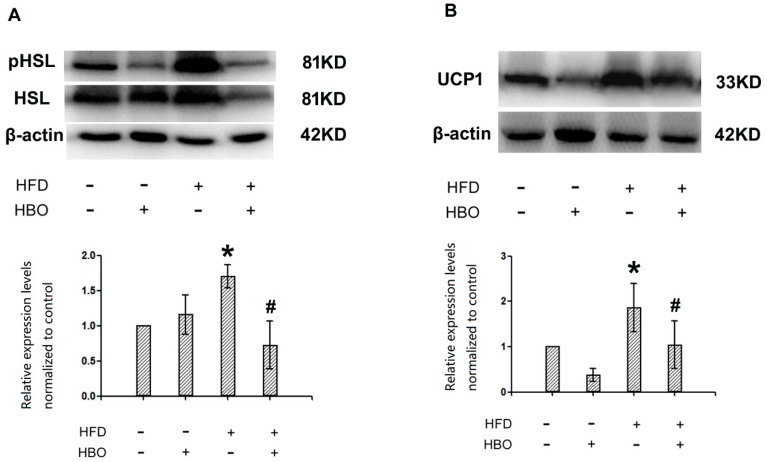
Western blotting for pHSL/HSL and UCP1 in brown adipose tissues. Brown adipose tissues from C57/B6 mice were homogenized in RIPA buffer with 1:100 PMSF and 1:100 phosphatase inhibitor cocktails, centrifuged at 14,000× *g* for 10 min, and the resulting supernatants were subjected to western blotting analysis for pHSL/HSL and UCP1. Images were acquired from a Vilber Lourmet gel imaging system and analyzed with ImageJ. Three samples from independent animals were included per group. Error bars represent standard derivation. (**A**): Representative blot images and quantifications of pHSL/HSL. (**B**): Representative blot images and quantifications of UCP1. *: statistically different from control group animals (*p* < 0.05). #: statistically different from HFD group animals (*p* < 0.05).

Western blotting results revealed that HFD treatment effectively enhanced the phosphorylation of HSL in brown adipose tissues, while HBO co-treatment returned the phosphorylation of HSL to normal level (Figure 8A). Similarly, UCP1 expression was remarkably enhanced in the brown adipose tissues from HFD treated animals, while HBO co-treatment abolished such changes (Figure 8B).

### 2.6. Western Blotting for CPT1b and PPARα in Skeletal Muscle

**Figure 9:**
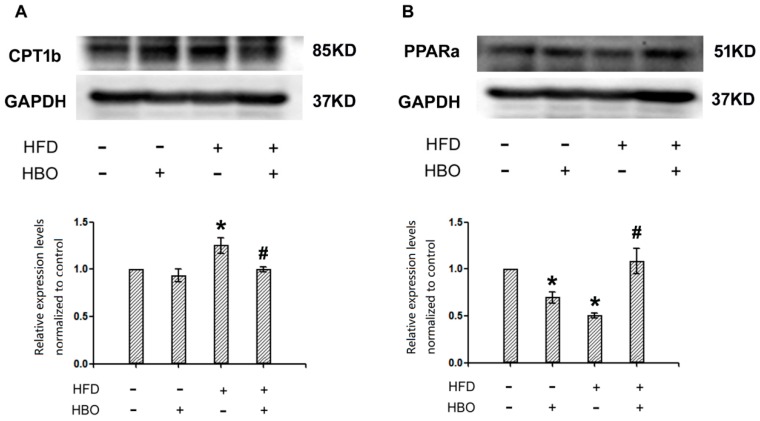
Western blotting for CPT1b and peroxisome proliferator-activated receptor alpha (PPARα) in skeletal muscle. Skeletal muscle samples from C57/B6 mice were homogenized in RIPA buffer with 1:100 PMSF, centrifuged at 14,000× *g* for 10 min, and the resulting supernatants were subjected to western blotting analysis for CPT1b and PPARα. Images were acquired from a Vilber Lourmet gel imaging system and analyzed with ImageJ. 3 samples from independent animals were included per group. Error bars represent standard derivation. (**A**): Representative blot images and quantifications of CPT1b. (**B**): Representative blot images and quantifications of PPARα. *: statistically different from control group animals (*p* < 0.05). #: statistically different from HFD group animals (*p* < 0.05).

Western blotting results indicated that HFD treatment significantly enhanced the expression of CPT1b relative to control, while HBO co-treatment brought the expression levels back to normal levels (Figure 9A). The enhanced expression of CPT1b following HFD treatment is likely compensation. Meanwhile, HFD treatment remarkably decreased the expression levels of PPARα, while HBO co-treatment reverted it back to normal (Figure 9B), suggesting that HFD treatment interrupted with beta oxidation, while HBO may exert its protective effects via protection of PPARα signaling. Interestingly, HBO treatment only seemed to decrease the expression levels of PPARα as well.

## 3. Discussion

Obesity and associated fatty acid metabolism dysfunction are major health issues across the world. Many efforts had been made towards effective methods for symptom alleviation and complication prevention, but current methods (pharmaceutical agents and physical therapies) are associated with adverse effects as well as limited efficacy. The mechanism of obesity is also being extensively investigated, with many candidates identified as potential therapeutic targets, among them, L-carnitine is a promising one. In the current study, the effects of HBO treatment against HFD-induced fatty acid metabolism dysfunction were investigated, HBO’s effect on endogenous levels of L-carnitine was investigated as the potential mechanism of action.

### 3.1. HBO and Fatty Acid Metabolism

HBO therapy has been utilized for multiple health conditions such as carbon monoxide poisoning, neurological damage, and radiation damage [26]. There is only limited evidence for its use in diabetes treatment [27], and it has not been approved to be used in obesity or fatty acid metabolism dysfunction. However, since HBO provides high level of oxygen, it is highly likely to facilitate fatty acid metabolism thus may serve as a potential therapy for such disorders. In previous studies, it had been reported that HBO treatment may improve metabolic capacity of the skeletal muscle [28] and decrease mouse body weight [29], suggesting that HBO may be beneficial in obesity and fatty acid metabolism dysfunction. Notably, these previous studies used either genetically modified animal model or chemically induced animal models, while the current study used diet-induced animal model, providing better relevancy. On the other hand, due to the potentially increased oxidative stress burden, higher level of HBO treatment (2.5 ATA) was also reported including liver damage [29], pulmonary edema, and maybe inflammation [30], while lower level of HBO treatment (2 ATA) did not induce such damages [31]. This phenomenon is mainly due to the nature of oxidative stress, in which a balance needs to be established, ensuring the necessary oxidation reactions in the organism to be performed effectively, while avoiding too many free radicals to be generated. In the current study, decreased body weight, less fat deposition and normal phosphorylation state of HSL (the major fatty acid mobilizing enzyme) in adipose tissues were observed in HFD-fed animals treated with HBO, which is consistent with the previous report [27], suggesting the potential of HBO to be used for obesity management. Regarding the potential adverse effects of HBO, HBO treatment without HFD indeed resulted in somewhat elevated serum FFA and brown adipose tissue weight, as well as increased fatty acid deposit in both white and brown adipose tissue. While none of these changes were statistically significant, it did indicate that hyperoxia may result in deleterious effects, especially in the organ systems not challenged with HFD. Interestingly, these endpoints all improved when animals received HFD, suggesting differential effects of HBO treatment depending on the diet.

### 3.2. Roles of L-Carnitine in HBO Mediated Protective Effects Against HFD-Induced Fatty Acid Metabolism Dysfunction

L-carnitine and its metabolites were known to exert protective effects in multiple organisms, such as the cardiovascular system, skeletal muscle, liver, and adipose tissue [32,33,34,35], probably due to their critical roles in fatty acid beta oxidation [36]. Focusing on fatty acid metabolism, L-carnitine supplementation was reported to decrease body weight in HFD-fed mice [37] and alleviate the pathological changes in adipose tissues [34]. However, few studies reported the relationship between HBO treatment and endogenous L-carnitine levels. HBO treatment was reported to improve fatty acid beta oxidation, which was reported to be mediated through enhanced glucose and lipid metabolism [28], which is closely associated with L-carnitine [38]. On the other hand, endogenous L-carnitine levels in circulation and in skeletal muscles were reported to be associated with fatty acid metabolism dysfunction: increased circulation L-carnitine levels were reported to be associated with metabolic syndrome [39], while lower skeletal muscle L-carnitine levels were reported to contribute to insulin resistance and metabolic syndrome as well [40]. In the current study, endogenous L-carnitine levels in both serum and skeletal muscle were measured. The results indicated that HFD induced higher circulation L-carnitine level, which is consistent with previous reports [39]. Slightly higher skeletal muscle L-carnitine levels were also observed, probably due to compensation. HBO treatment decreased circulating L-carnitine levels while increased skeletal muscle L-carnitine levels, suggesting that L-carnitine is involved in HBO-mediated protection against fatty acid metabolism dysfunction. It seems like HBO facilitates beta oxidation in skeletal muscle, just as reported by Takemura et al. [41]. Our study made one step further focusing on the effects of HBO on circulating and skeletal L-carnitine contents. HBO treatment seemed to redistribute endogenous L-carnitine, translocating L-carnitine from circulation to skeletal muscle. While further investigation is still needed, this is a good indicator that L-carnitine is critical for HBO treatment in obesity/fatty acid metabolism dysfunction.

### 3.3. L-Carnitine and PPARα/CPT1b

PPARα is a classical key regulator of fatty acid metabolism, which regulates several key enzymes in fatty acid beta oxidation, such as ACOX1, FABPs and CPT1 [25,42,43]. Although the strongest expression of PPARα was observed in the liver, it is also expressed in the heart and skeletal muscles, and regulates fatty acid metabolism in these striated muscle tissue as well [44,45,46]. In skeletal muscle, it has been observed that PPARα, not PPARδ mediates responses to PPAR agonists [47]. PPARα and L-carnitine are both critical participants in fatty acid beta oxidation, and can often interact with each other: PPARα agonists may decrease serum L-carnitine levels [48], while L-carnitine supplementation may enhance PPARα expression [49]. Cooperative interactions between L-carnitine and PPARα were also reported [50]. In the current study, PPARα expression was significantly depressed by HFD challenge, which is consistent with a previous report [51]. HBO treatment effectively restored the expression levels of PPARα in the skeletal muscle, suggesting that HBO’s protective effects may at least partially be mediated through the restoration of PPARα signaling. Notably, the expression levels of PPARα in animals treated with only HBO also decreased, although the average value is still higher than those with HFD. This result is consistent with observed serum FFA level changes and adipose tissue pathological changes, suggesting that HBO treatment should be cautiously applied on metabolically normal individuals.

Among the PPARα downstream molecules, CPT1 is the rate-limiting enzyme for the carnitine palmitoyltransferase system, connecting fatty acid acyl groups to carnitine. Interruption of CPT1 is frequently associated with HFD induced metabolic dysfunction and diabetes [52,53]. Furthermore, the function of CPT1 is directly linked to the endogenous levels of L-carnitine, thus it is highly likely to be affected when L-carnitine levels change [54]. In the current study, CPT1b (skeletal muscle isoform) expression levels remarkably elevated in HFD-treated animals, while HBO treatment returned it to normal levels. This result is consistent with Leduc-Gaudet et al. [55], in which HFD increased CPT1b expression levels in skeletal muscles. Another study reported no statistical difference in CPT1b expression levels following HFD, but fenofibrate, a PPARα agonist significantly decreased its expression [56]. In the current study, HBO treatment both enhanced PPARα expression and decreased CPT1b expression. One possible explanation is that other molecules modulates CPT1b under HFD challenge. When HBO treatment recovered PPARα signaling, such changes were reverted. Another possibility is that the actual activity of PPARα/CPT1b is different from their expression levels. Assays of PPARα and CPT1b activities under HFD and/or HBO treatment have been planned. Our results suggest that HBO may enhance PPARα expression in skeletal muscle in a pattern similar to PPARα agonists, which is likely part of its mechanism of action in protecting against HFD induced fatty acid metabolism dysfunction and obesity.

### 3.4. Limitations of the Current Study and Future Directions

**Figure 10:**
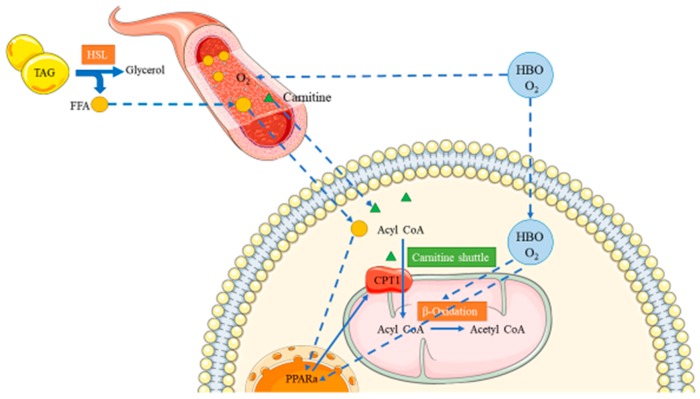
Proposed mode of action. TAG: triacylglycerol. HSL: hormone sensitive lipase. FFA: free fatty acid. HBO: hyperbaric oxygen. CPT1: carnitine palmitoyltransferase 1. PPARα: peroxisome proliferator-activated receptor alpha.

The current study revealed the protective effects of HBO treatment against HFD-induced fatty acid metabolism dysfunction/obesity, as well as the roles of L-carnitine and associated PPAR signaling. The proposed mode of action of HBO and L-carnitine was reported in Figure 10. However, interventions such as extraneous L-carnitine supplementation and PPARα agonist/antagonist treatments were absent due to the limited capacity of animal housing and handling. These experiments are guaranteed in the future studies.

## 4. Materials and Methods

### 4.1. Materials

L-carnitine standard (C0158, CAS 541- 15-1) was purchased from Sigma Aldrich (St. Louis, MO, USA). Primary antibody against p-HSL/HSL, CPT1b and PPARα/UCP1 were purchased from Cell Signaling Technology (Shanghai, China), Bioss (Beijing, China), and Abcam (Shanghai, China), respectively. Antibody against GAPDH and β-actin were purchased from ZSGB-BIO (Beijing, China) and Cell Signaling Technology (Shanghai, China), respectively. Hematoxylin-eosin staining kit was purchased from Beyotime (Beijing, China). Immunohistochemistry kit was purchased from ZSGB-BIO (Beijing, China). FFA measurement kit was purchased from Solarbio (Beijing, China). Other general laboratory supplies were all of the highest grade obtainable.

### 4.2. Animal Housing, Treatment and Sample Collection

C57/B6 mice were purchased from Qingdao Institute of Drug Control. Upon arrival, animals were kept in 21–25 °C environment with 12-h light/dark cycle. Food and water were provided Ad libitum. After one-week adaptation, animals were randomly assigned into control, HFD, HBO treatment, and HFD + HBO treatment groups. Six animals were included for each group. Control and HBO treatment animals received normal chew diet, while HFD and HFD + HBO treatment animals received 40% HFD (15% Lard, 3% soybean oil, 5% egg yolk, 18% sugar, and 59% chow diet) for ten weeks. Then, HBO treatment and HFD + HBO treatment animals were subjected to hyperbaric treatment (5 min pressure rise stage, 60 min stabilization stage with 2.0 atmospheres absolute and 100% oxygen, 5 min depression stage) [57] for four more weeks, in which the diet treatments continued. Please refer to Figure 1A for the timeline of the study. The 12-h cumulative food intake was measured after the first HBO treatment session, in which HFD had elevated food intake as expected, but HBO had no significant effects (Supplementary material 2). At the end of the 14-week study, animals were sacrificed under anesthesia (33 mg/kg pentobarbital, intraperitoneal injection). Serum, skeletal muscle, EWAT, IWAT and BAT tissues were collected and stored either in −80 °C freezer or fixed in 4% paraformaldehyde for later use. All the procedures used in this study have been approved by the Qingdao University Animal Care and Use Committee in keeping with the National Institutes of Health guidelines (Approve code: 20190603).

### 4.3. Histological Methods

After fixing in 4% formaldehyde for 24 h, the EWAT and BAT tissues were histologically processed and embedded in paraffin as described in [58] with minor modifications. The paraffin blocks were then sectioned on a microtome (Leica RM2016, Leica, Germany) at 6-micron thickness. The sections were dried in oven at 37 °C overnight, and then subjected to hematoxylin and eosin staining or immunohistochemistry following manufacturer’s instructions. The primary antibody dilution for the HSL antibody was 1:50. ImageJ (NIH, US) was used to semi-quantify the fraction of positively stained area. Three independent experiments were performed per group.

### 4.4. Serum FFA Level Measurement

The serum FFA levels were determined with a serum FFA measurement kit (BC0595, Solarbio, CN) following manufacturer’s instructions. A standard curve was established along with actual samples. After data acquisition with a plate reader (M5, MD-SpectraMax, Molecular Devices, San Jose, CA, USA) at 550 nm absorption, the serum concentration of FFA was calculated according to the standard curve.

### 4.5. Liquid Chromatography-Tandem Mass Spectrometry Measurement for Serum and Skeletal Muscle L-Carnitine Levels

Endogenous L-carnitine levels in serum and skeletal muscle were measured with liquid chromatography-tandem mass spectrometry (LC/MS-MS). LC–MS/MS was performed by Beijing Mass Spectrometry Medical Research Co.,Ltd. (Beijing, CN). For the serum samples, 20 uL serum was mixed with 100 uL protein precipitant thoroughly, centrifuged at 13,200 rpm for 4 min. For the skeletal muscle samples, appropriate amount of distilled water was added to the samples, homogenized and centrifuged at 13,200 rpm for 10 min. Fifty microliters of supernatant was then mixed with 100 uL protein precipitant and centrifuged at 13,200 rpm for 4 min. The resulting samples were then subjected to LC–MS/MS (Ultimate3000—API 3200 Q TRAP). For the detailed parameters used in the LC–MS/MS measurement and the quality controls, please refer to the supplementary material.

### 4.6. Western Blotting

Western blotting for PPARα and CPT1b expression levels in skeletal muscles as well as for UCP1, pHSL and HSL in brown adipose tissues were performed as described in [59] with minor modifications. Briefly, samples were homogenized in RIPA buffer with 1:100 phenylmethylsulfonyl fluoride and 1:100 phosphatase inhibitor cocktail (Epizyme, China, GRF102) added and centrifuged at 14,000× *g* for 10 min. The resulting supernatants were subjected to BCA assay for protein concentrations. Equal amounts of protein were mixed with sampling buffer and denatured at 95 °C for 5 min, and then subjected to SDS-PAGE electrophoresis. After transferring proteins to PVDF membrane and blocked with non-fat milk, primary antibodies (1:1000 for PPARα, CPT1b, UCP1, pHSL, and HSL) and 1:5000 for GAPDH or β-actin) were used to probe for the target proteins, and the bands were visualized by ECL system with a Fusion Solo S gel imaging system (Vilber Lourmat, Collégien, France). ImageJ (NIH, US) was used to semi-quantify the band densities. Three independent experiments were performed per target.

### 4.7. Statistics

Statistical analysis was performed with SPSS 17.0. The body weights at 0 week and 10 weeks were analyzed with one way analysis of variance (ANOVA), and all the other data were analyzed with two by two factorial design ANOVA. Results were considered statistically significant when *p* < 0.05.

## 5. Conclusions

In the current study, HBO treatment may alleviate HFD-induced fatty acid metabolism dysfunction/obesity in C57/B6 mice, which seems to be associated with corrected circulation and skeletal muscle L-carnitine levels and PPARα expression levels. HBO treatment is a promising physical therapy for management of metabolic syndrome and obesity.

## Supplementary Materials

The following are available online at https://www.mdpi.com/1420-3049/25/1/176/s1.
