# Supplementary material for: L-Carnitine Is Involved in Hyperbaric Oxygen-Mediated Therapeutic Effects in High Fat Diet-Induced Lipid Metabolism Dysfunction
Source: Molecules. 2020 Jan 1;25(1):176. doi: 10.3390/molecules25010176 (PMC6982999; doi:10.3390/molecules25010176)
Supplement: Supplementary file 1 [file molecules-25-00176-s001.zip › Supplementary material 1.pdf]

LC-MS/MS Parameters

LC parameters:

Column: MSLab HP-C18（100*4.6mm 5um）

Temperature: 50 ºC

Flow velocity: 1 ml/min

Mobile Phase (A): Water (0.1% formic acid)

Organic Phase (B): Acetonitrile (0.1% formic acid)

Sample volume: 5 ul

Gradient：

	1	2	3	4	5	6	7	8
Retention	0.0	1.0	1.1	5.0	5. 1	8.0	8. 1	12.0
%A	90	90	50	50	0	0	90	90
%B	10	10	50	50	100	100	10	10

MS Parameters:

Electrospray ionization, positive ion mode (+ESI)

Multiple reaction monitoring (MRM)

CUR:20psi

CAD: Medium

IS: +5500V

TEM:500°C

GS1:55 psi

GS2: 60 psi

EP:10

CXP:2.0

MRM Parameters

4.3 MRM

162.100/103.100 Da

LC-MS/MS Quality Control

Accuracy

Accuracy is calculated with the following equation:

(Calculated concentration - Analyte Concentration)/Analyte Concentration.

Sample Name	Sample Type	Analyte Concentration (ng/mL)	Calculated Concentration (ng/mL)	Accuracy (%)
S1	Standard	125	130	104
S2	Standard	250	249	99.5
S3	Standard	500	478	95.7
S4	Standard	1000	992	99.2
S5	Standard	2000	2030	101
QC1	Quality Control	200	192	96
QC2	Quality Control	800	798	99.7
QC3	Quality Control	200	198	99.1
QC4	Quality Control	800	791	98.9

S1-S5: standard curves

QC1, QC2: Quality control samples prior to actual sample measurements.

QC3, QC4: Quality control samples after actual sample measurements.

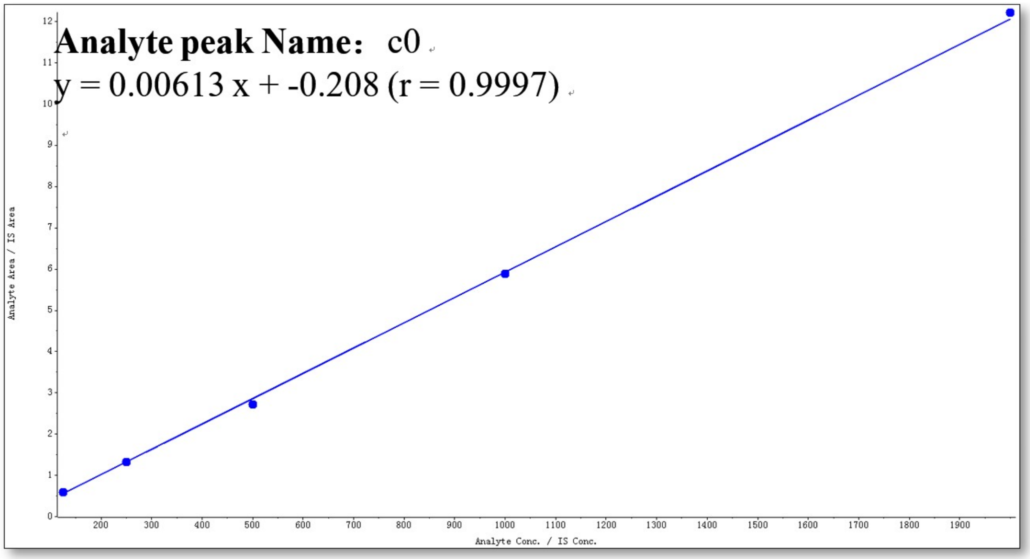

Standard curve for the serum samples

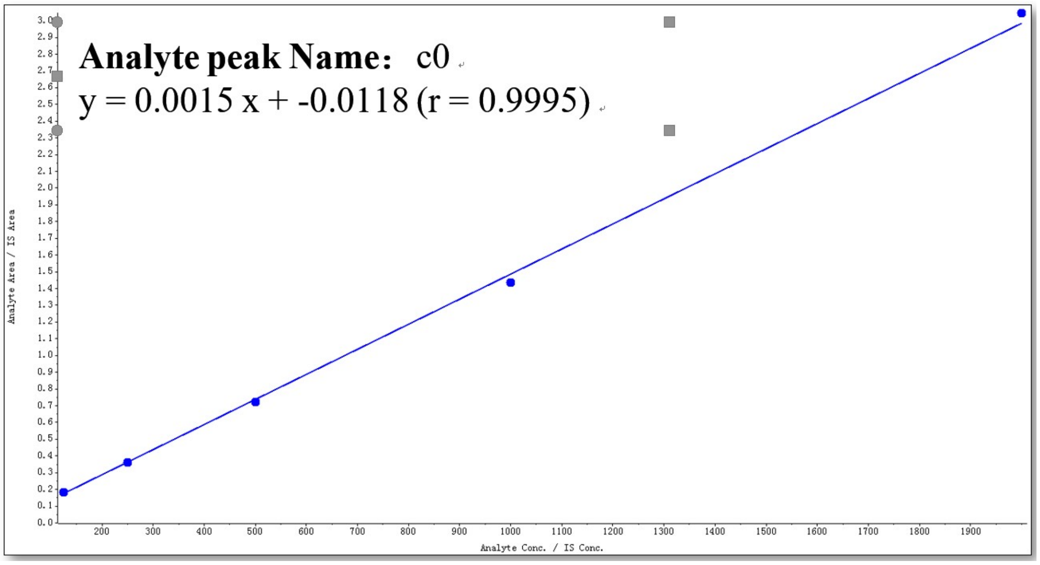

Standard curve for the skeletal muscle samples.
