# Supplementary material for: L-Carnitine Is Involved in Hyperbaric Oxygen-Mediated Therapeutic Effects in High Fat Diet-Induced Lipid Metabolism Dysfunction
Source: Molecules. 2020 Jan 1;25(1):176. doi: 10.3390/molecules25010176 (PMC6982999; doi:10.3390/molecules25010176)
Supplement: Supplementary file 1 [file molecules-25-00176-s001.zip › Supplementary material 2.pdf]

Supplementary Material 2

12-h cumulative food intake test after the first HBO session

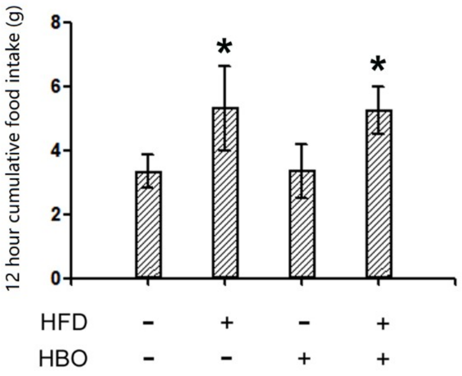

C57/B6 mice were kept in 21–25°C environment with 12-h light/dark cycle. Food and water were provided ad labium. Animals received normal chew diet, or HFD (15% lard, 3% soybean oil, 5% egg yolk, 18% sugar and 59% chew diet) for a total of 14 weeks. HBO treatment (5 min pressure rise stage, 60 min stabilization stage with 2.0 atmospheres absolute and 100% oxygen, 5 min depression stage) were applied once, and then the 12-h cumulative food intake was tested. Briefly, animals underwent 12-h of food deprivation, and then new food (standard mouse chew for control and HBO only animals, and high fat diet food for HFD and HFD+HBO animals) were provided for 12 hs, then all the remaining food were weighed with an electronic scale (TE412-L; Satorius, Göttingen, Germany). 3 animals per batch were tested. Error bars represent standard derivation. *: statistically different from control animals.
